# Chemical Arsenal for the Study of *O*-GlcNAc

**DOI:** 10.3390/molecules16031987

**Published:** 2011-02-28

**Authors:** Eun J. Kim

**Affiliations:** Department of Science Education-Chemistry Major, Daegu University, Gyeongbuk 712-714, Korea; E-Mail: eunkim@daegu.ac.kr; Tel.: +82-53-850-6981; Fax: +82-53-850-6989

**Keywords:** phosphorylation, *O*-GlcNAcylation, OGT, OGA, chemical tools

## Abstract

The concepts of both protein glycosylation and cellular signaling have been influenced by *O*-linked-β-*N*-acetylglucosamine (*O*-GlcNAc) modification (*O*-GlcNAcylation) on the hydroxyl group of serine or threonine residues. Unlike conventional protein glycosylation, *O*-GlcNAcylation is localized in the nucleocytoplasm and its cycling is a dynamic process that operates in a highly regulated manner in response to various cellular stimuli. These characteristics render *O*-GlcNAcylation similar to phosphorylation, which has long been considered a major regulatory mechanism in cellular processes. Various efficient chemical approaches and novel mass spectrometric (MS) techniques have uncovered numerous *O*-GlcNAcylated proteins that are involved in the regulation of many important cellular events. These discoveries imply that *O*-GlcNAcylation is another major regulator of cellular signaling. However, in contrast to phosphorylation, which is regulated by hundreds of kinases and phosphatases, dynamic *O*-GlcNAc cycling is catalyzed by only two enzymes: uridine diphospho-*N*-acetyl-glucosamine:polypeptide β-*N*-acetylglucosaminyl transferase (OGT) and β-D-*N*-acetylglucosaminidase (OGA). Many useful chemical tools have recently been used to greatly expand our understanding of the extensive crosstalk between *O*-GlcNAcylation and phosphorylation and hence of cellular signaling. This review article describes the various useful chemical tools that have been developed and discusses the considerable advances made in the *O*-GlcNAc field.

## 1. Introduction

**Figure 1:**
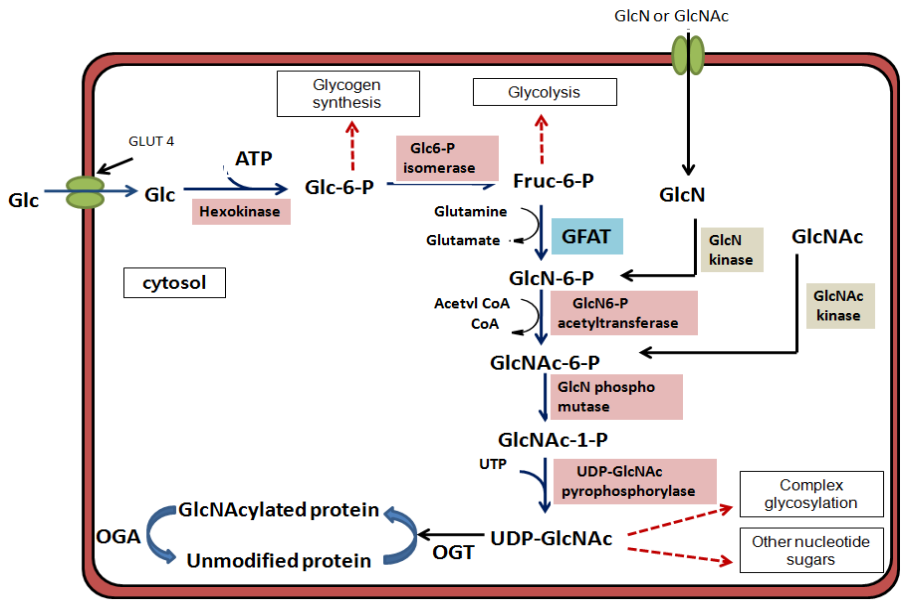
The hexosamine biosynthetic pathway (HBP).

Until the mid 1980s, glycosylation was thought to occur on the cell surface proteins and luminal face of secreted proteins, rather than on nuclear and cytosolic proteins. In 1984, a new localization of glycosylation was first observed during studies designed to detect terminal *O*-linked *N*-acetylglucosamine (*O*-GlcNAc) on the surfaces of living lymphocytes [1]. This glycosylation predominantly occurs on proteins within the nucleus and cytoplasm. The structure of this new glycosylation found on nucleocytoplasmic proteins was identified as the single *O*-GlcNAc. Unlike conventional glycosylation occurring within the secretory pathway, this new type of intracellular glycosylation has the following three unique features: (1) It occurs exclusively on cytoplasmic and nuclear proteins, rather than on the extracellular and luminal localization; (2) It is generally not elongated to more complex structures; (3) It is a dynamic modification. It can be removed from and reinstalled on the polypeptides more rapidly than the protein is turned over [2,3] and its cycling “on and off” is in response to different physiological stimuli, such as nutrients, hormones, growth factors, and stress [4,5,6,7]. This stimuli-responsive dynamic nature of *O*-GlcNAc modification, which has been termed “*O*-GlcNAcylation”, resembles phosphorylation, which is a common posttranslational modification (PTM) of nuclear and cytosolic proteins. Although these two PTMs share similar properties, they are regulated very differently. Phosphorylation is regulated by over 500 kinases and 140 phosphatases [8], whereas only two enzymes are involved in the regulation of *O*-GlcNAc cycling in humans. These two enzymes are a glycosyltransferase termed as *O*-GlcNAc transferase (OGT) [9,10,11] and a glycoside hydrolase termed as *O*-GlcNAcase (OGA or formally known as hexosaminidase C [Hex C]) [12,13]. OGT installs the sugar moiety (GlcNAc) on target proteins using the uridine5’-diphosphate (UDP)-GlcNAc substrate which is a high-energy metabolite synthesized via the hexosamine biosynthetic pathway (HBP) (Figure 1). A reverse enzyme, OGA returns glycosylated proteins to their unmodified state by catalyzing the removal of *O*-GlcNAc. The level of UDP-*N*-acetylglucosamine (UDP-GlcNAc) and the extent of protein GlcNAcylation appear to be sensitive to nutrient (*i.e.*, glucose, glucosamine and fatty acid) availability [5,14,15].

Interestingly, proteins known to be modified with *O*-GlcNAc can also be phosphorylated, which implies a dynamic interplay between *O*-GlcNAcylation and phosphorylation [16]. In some proteins, *O*-GlcNAc competes with phosphate for the same site and results in the change of the activity or stability of the proteins. For example, the Ser16 residue of estrogen receptor β (ER-β) can be modified both with *O*-GlcNAc and *O*-phosphate in a reciprocal manner [17,18,19]. This site of ER-β is within a region that is related to protein degradation. Site-directed mutagenesis studies showed that the phosphorylation of ER-β at this site increases the transcriptional activity, but induces rapid degradation, while *O*-GlcNAcylation at Ser16 increases its stability, but decreases its transcriptional activity [18]. Another example of reciprocal interplay between *O*-GlcNAcylation and phosphorylation at the same residue can be found in endothelial nitric oxide synthase (eNOS) [20]. eNOS is reciprocally modified with *O*-GlcNAc and phosphate at Ser1177. Phosphorylation of eNOS by protein kinase B (PKB or AKT) at this site activates the enzyme and positively regulates penile erection. However, in diabetic patients, *O*-GlcNAcylation at Ser1177 prevents activation of eNOS by AKT, which contributes to erectile dysfunction [20].

In some cases, *O*-GlcNAc and phosphate modification occur at proximal sites in the reciprocal relationship. An example is the tumor suppressor p53 [21], which contains two modification sites at Ser149 and Thr155 for *O*-GlcNAcylation and phosphorylation, respectively, and shows a reciprocal relationship between these two proximal sites. *O*-GlcNAcylation of p53 at Ser149 reduces the phosphorylation of the nearby site (Thr155), which subsequently prevents the interaction between Mdm2, an E3 ubiquitin ligase, and p53, a prerequisite for proteasomal degradation.

Therefore, an increase in *O*-GlcNAcylation of p53 at Ser149 reduces p53 ubiquitination and thereby stabilizes the protein p53 [21]. The second example of proximal site competition can be found in calcium/calmodulin-dependent kinase IV (CaMKIV). This enzyme has several *O*-GlcNAcylated and phosphorylated sites and regulates downstream protein activities in response to the intracellular Ca^2+^ concentrations [22]. Phosphorylation of CaMKIV at Thr200 increases its kinase enzymatic activity, but *O*-GlcNAcylation at Ser189 results in a reciprocal decrease of its phosphorylation at Thr200 and inhibits the enzyme activity. Structural analysis revealed that this *O*-GlcNAc site is located within the adenosine triphosphate binding region of the enzyme [22]. In addition to the reciprocal relationship between these two modifications at the same or proximal sites of the proteins discussed above, they also exhibit an interplay among distantly located sites on proteins such as insulin receptor substrate 1 (IRS-1). When phosphorylation occurs on several specific serine residues (Ser307, 612, 632/635), it has a negative effect on insulin signaling, whereas phosphorylation on Ser302 and Ser629 positively regulates insulin signaling [23]. *O*-GlcNAcylation sites of IRS-1 are found in distantly located regions such as Ser914, Ser1009, Ser1036, Ser1041 and the global increase in *O*-GlcNAcylation has been shown to exert a negative regulatory effect on insulin signaling [24,25]. These observations suggest that these two PTMs on IRS-1 affect each other’s regulation both reciprocally and synergistically. It is now believed that protein *O*-GlcNAcylation participates in many fundamental aspects of cellular homeostasis such as cell signaling, mRNA transcription, and protein stability [26,27] and acts as a common cellular regulator by interacting with *O*-phosphorylation reciprocally or synergistically [28,29,30]. Dysregulation of *O*-GlcNAc cycling has been related to various diseases, including diabetes [24,31,32], cancer [21,33] and neurodegenerative disorders [34,35]. The roles of nutrient responsive *O*-GlcNAc cycling in the embryonic development and epigenetic programming of developmental fate have also emerged [36]. Since its first discovery, a variety of methods that detect and modulate *O*-GlcNAc cycling has been developed [37,38,39,40,41]. Armed with these tools, the diverse set of *O*-GlcNAc-modified proteins have been identified [42] and the functional roles of *O*-GlcNAc cycling have matured. This review highlights recent advances in the development of useful chemical tools that have been applied to elucidate the molecular basis of biological processes in which *O*-GlcNAc participates. The chemical tools designed for *O*-GlcNAc proteomics and the monitoring of the activities of enzymes involved in *O*-GlcNAc cycling. Since the inhibitors of these enzymes have been described in the two most recent review articles [43,44], the topic of enzyme inhibitors is briefly discussed.

## 2. Chemical Tools for *O*-GlcNAc Proteomics

The major impediment to determining the molecular mechanistic roles of *O*-GlcNAc in specific signaling cascades and in transcriptional regulation has been the lack of sensitive and easy-to-use tools for identifying *O*-GlcNAcylated proteins and site localization. Mapping the modification site(s), however, is a particularly challenging task due to its two special characteristics: (1) The stoichiometry of *O*-GlcNAc modification at each site on proteins is usually low; (2) The glycosidic linkage between the GlcNAc moiety and Ser/Thr is chemically and enzymatically labile. In early studies, mapping *O*-GlcNAc site(s) of the protein began with the purification of the target protein. GlcNAc moieties of the purified protein were then enzymatically tagged with radiolabeled galactose using radiolabeled UDP-Gal and bovine milk β-1,4-galactosyltransferase (GalT1) [1]. The radiolabeled proteins were subject to proteolysis resulting in a mixture of tryptic peptides. The tryptic peptides that contain radioactivity were purified using high performance liquid chromatography (HPLC). Then the purified peptide sequence was determined by automated Edman degradation and the residue containing the radioactivity was elucidated by manual Edman degradation [45]. Although several *O*-GlcNAc sites of some proteins such as neurofilament H [46], L and M [47], estrogen receptor [48,49], and synapsin I [50] have been mapped using this method, this method has two drawbacks: (1) The whole process consists of many time-consuming steps containing several rounds of HPLC purification and Edman degradation steps for identification of modification sites; (2) Due to the low detection limits of radiolabeled galactose, this method needs large amounts of expensive radioactive sugar nucleotide.

Mass spectrometric (MS) analysis has been applied to identify many *O*-GlcNAcylated proteins. However, determination of the site modification remains difficult due to the labile property of the glycosidic linkage between Ser/Thr residues of peptides and *O*-GlcNAc, as mentioned before. The *O*-GlcNAc moiety falls off from the peptide/protein in a mass spectrometer upon fragmentation by standard collision-induced dissociation (CID) or collision-activated dissociation (CAD), resulting in the loss of any site information. Furthermore, in MS, if a small portion of unmodified peptidesco-exist, unmodified peptides are preferentially ionized, which suppresses the signal of the glycopeptides. As stated above, proteins known to be modified with *O*-GlcNAc are not usually 100% glycosylated. Therefore, peptides generated from a protein sample are mostly in the unmodified state, which can severely suppress the signal of the corresponding *O*-GlcNAcylated peptides. Thus, it is necessary to isolate or enrich *O*-GlcNAcylated proteins/peptides from other non-glycosylated species. Several enrichment strategies that allow for the efficient isolation of molecules of interest from other species have been developed. A general approach for the *O*-GlcNAc proteomic study includes three steps: (1) Detection and enrichment of O-GlcNAcylated proteins; (2) Identification of *O*-GlcNAc-modified proteins; (3) Identification of the modification site(s).

### 2.1. *O*-GlcNAc Enrichment Strategies

#### 2.1.1. Conventional antibody/lectin-based enrichment approaches

The simplest biological approaches for *O*-GlcNAc enrichment utilize either *O*-GlcNAc antibodies or *O*-GlcNAc-binding lectin, which can be conjugated to an epitope tag for visualization and forsolid-phase-based purification [51,52,53]. Two *O*-GlcNAc antibodies are commonly used: RL2 [54], which is produced against the nuclear pore protein complex fraction, and carboxyl terminal domain (CTD) 110.6 [55], which is produced against the synthetic *O*-GlcNAcylated peptide based on the RNAP II CTD. However, the binding affinity of antibodies to *O*-GlcNAc is relatively low, which prevents the stringent washing conditions required for reducing nonspecific interactions. Commonly used *O*-GlcNAc binding lectin is wheat-germ agglutinin (WGA). However, WGA is not specific for a particular sugar. It can bind not only *O*-GlcNAc monosaccharide but also oligosaccharides containing terminal *N*-acetylglucosamine or chitobiose. Furthermore, WGA binds weakly to monosaccharide *O*-GlcNAc. Therefore, the use of these probes suffers from the contamination with nonspecific binding partners. In addition, since all of these probes bind preferentially with high abundance proteins or those with multiple clustered *O*-GlcNAc residues, the detection and isolation of low abundance proteins with single *O*-GlcNAc are often elusive. Indeed, these probes failed to detect the *O*-GlcNAc moiety onα-crystallin that has only one modification site [56,57] and has low stoichiometry of glycosylation (<10%) [56,57,58]. Nevertheless, using WGA lectin-based enrichment approach in combination with MS techniques a number of new proteins involved in synaptic transmission have been identified [52,59].

#### 2.1.2. Metabolic labeling of *O*-GlcNAc-based enrichment approaches

**Figure 2:**
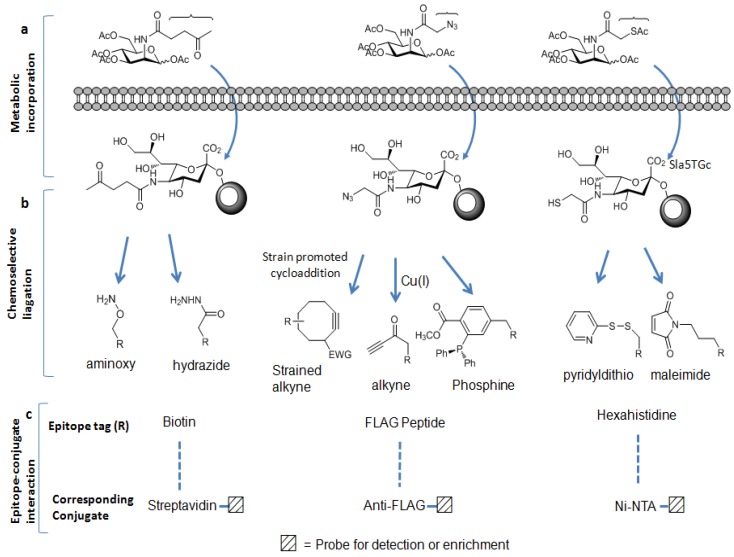
Metabolic labeling carbohydrate-based enrichment strategies: (a) chemical handles can be displayed on the glycoproteins; (b) these chemical handles can be chemoselectively ligated to the appropriate epitope tags;and (c) by the epitopes-conjugates interactions, labeled glycoproteins can be isolated and enriched.

Metabolic carbohydrate engineering strategies have been developed as complementary methods to biological antibody/lectin-based enrichment approaches. These strategies were originated by Reutter *et al.* to engineer the cell surface carbohydrates [60,61]. In these approaches, slightly modified monosaccharide containing a specific functional group termed as “chemical handle” is used as an alternative substrate to natural sugar substrate. This unnatural monosaccharide is taken up by the cells and incorporated into the cellular glycoconjugates by the biosynthetic machinery of the cell. Ketone [62,63], azide [64,65,66,67], and thiol [68] functional groups have been used as the chemical handles to metabolically label glycoproteins in mammalian cells (Figure 2a).

Once incorporated into cellular glycoconjugates, the chemical handle can be ligated to either a visualization tag or an enrichment tag through chemoselective reaction between the chemical handle and an appropriate reagent probe [69,70]. The ketone moiety is reactive to amino groups such as aminoxy or hydrazide reagent. The azide group can selectively react with a terminal alkyne-containing reagent or with a phosphine reagent, whereas the thiol moiety readily reacts with the disulfide-bridged reagent or maleimide reagent. Among these chemical handles, the azide moiety has proved to be one of the most useful chemical handles since it satisfies the following requirement of bioorthogonality: It is absent from biological systems and is unreactive with endogenous biological functional groups. However, it has unique reactivity with phosphine- and alkyne-based probes [71] as mentioned before. Furthermore, its small size induces minimum perturbation of substrate structure and it is stable within cellular glycoconjugates. The most commonly employed bioorthogonal reactions with azides include the Staudinger ligation [64] with phosphine probes, and the copper (I)-catalyzed cycloaddition with terminal alkyne probes (click chemistry) [72,73]. More recently copper-free click chemistry has been developed [74,75]. Unlike the conventional click chemistry labeling method, this new method does not require a cytotoxic metal catalyst because alkyne generated *in situ* is highly strained enough to promote rapid cycloaddition with azide to form a stable triazole conjugate (Figure 2b). Using these methods, azido-labeled biomolecules can be covalently ligated to the appropriate reagents having epitope tags [70,76] such as the FLAG peptide, biotin, or hexahistidine (His6). Utilizing these epitope tags, azide-labeled biomolecules can be detected and enriched by the use of anti-FLAG antibody, streptavidin, or nickel-nitrilotriacetic acid (Ni-NTA) derivative conjugate, respectively (Figure 2c) [69,70,77]. Due to its very strong affinity for streptavidin, biotin has been chosen as the most popular epitope tag for detection and affinity purification. This strong biotin-streptavidin interaction can tolerate harsh washing conditions, allowing the removal of nonspecifically bound materials, thereby minimizing contamination from unmodified peptides and decreasing the ion suppression effect in the MS analysis. In contrast, the other epitope tags are not amenable to such stringent washes.

**Figure 3:**
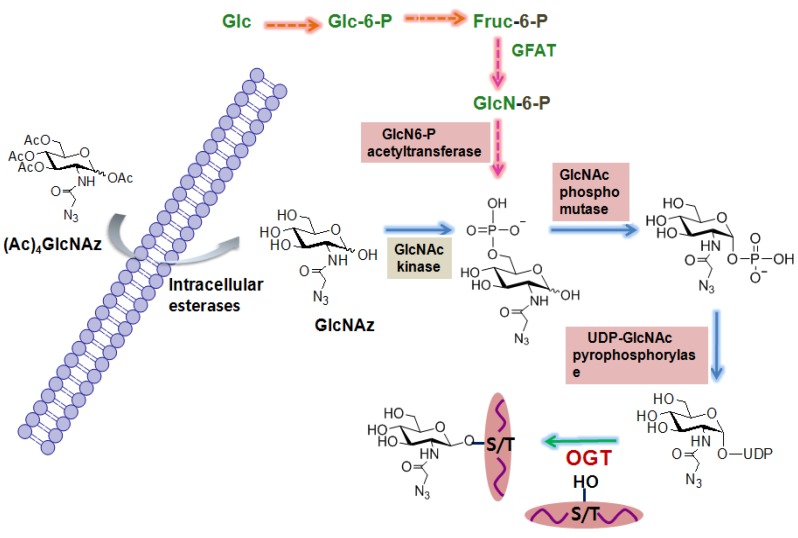
Metabolic labeling of carbohydrate strategies for the global identification of *O*-GlcNAc-modified proteins.

The first *O*-GlcNAc proteomic study using these metabolic carbohydrate engineering strategies was performed by Vocadlo *et al.* using azide-containing GlcNAc analog (GlcNAz) to globally characterize *O*-GlcNAcylated proteins in cells. The general procedure that has been widely used is described as follows (Figure 3). In the first step, cells are incubated with peracetylated GlcNAz. Peracetylation of this unnatural sugar facilitates its uptake by living cells because it is expected to penetrate more efficiently the cell surface membrane due to its hydrophobic nature. Upon its cellular entry, the peracetylated GlcNAz is rapidly deacetylated by intracellular esterases. Then GlcNAz enters the hexosamine salvage pathway just like glucosamine, and is converted into UDP-*N*-azidoacetylglucosamine (UDP-GlcNAz). OGT utilizes UDP-GlcNAz and attaches GlcNAz to serine or threonine site(s) of proteins [66]. GlcNAz-labeled proteins are then conjugated to biotinylated phosphine via the Staudinger ligation and the resultant biotinylated proteins are affinity-purified using streptavidin beads. Proteins bound to the beads are digested with trypsin and tryptic peptides are analyzed by MS analysis. The major disadvantage of this metabolic labeling approach is that it can perturb metabolic pathways of cells, and thus the captured glycosylation state of proteins may not be physiologically relevant. In addition, GlcNAz can be incorporated into the cell surface glycoproteins, although the efficiency of its incorporation is low, resulting in a false positive assignment as the *O*-GlcNAc-modified proteins. Nevertheless, this method has been widely used to globally identify *O*-GlcNAc-modified proteins ranging from zebrafish [78] to human cancer cells [79].

#### 2.1.3. Chemoenzymatic tagging of *O*-GlcNAc-based enrichment approach

**Figure 4:**
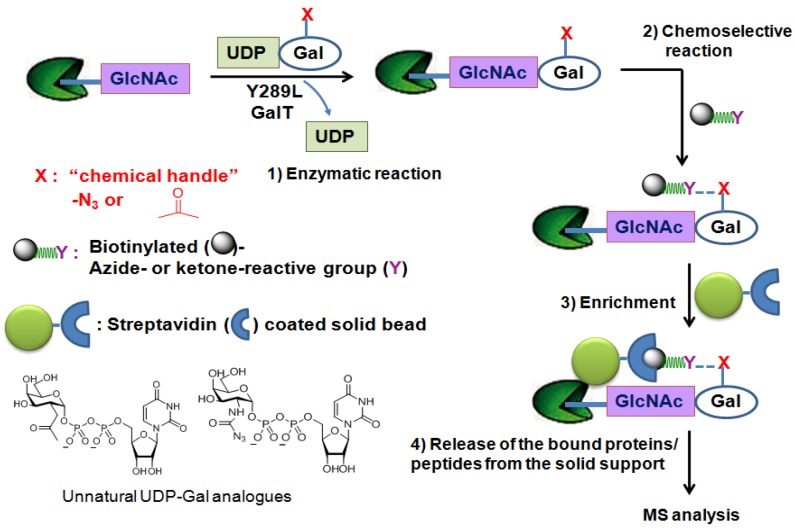
Chemoenzymatic tagging of *O*-GlcNAc-based enrichment strategies.

In an attempt to directly characterize physiologically relevant *O*-GlcNAcylated proteins, the chemoenzymatic approach has been applied. This method uses a genetically engineeredβ-1,4-galactosyl transferase (Y289L GalT mutant) [80,81] and an unnatural UDP-galactose analogue as a sugar-donor substrate that contain a chemical handle such as the azido [82] or ketone moiety [83] to label *O*-GlcNAcylated proteins/peptides. The enlarged donor-substrate binding pocket of this Y289L GalT mutant enables it to efficiently transfer unnatural galactose to the C-4 hydroxyl group of GlcNAc in glycoproteins/glycopeptides. Once transferred, the azido- or ketone-containing galactose label of GlcNAc can be subjected to chemoselective ligation with a suitable reagent having biotin (Figure 4). Then, the biotin-labeled proteins/peptides can be enriched through streptavidin affinity chromatography and subjected to the MS analysis. Using this approach, several low abundance *O*-GlcNAcylated proteins (c-Fos, c-Jun, ATF-1, and CBP) and two modification sites on OGT were identified [84].

As noted above, the strong interaction between biotin and streptavidin has made the biotin molecule the most popular epitope tag for the detection and enrichment of molecules of interest. However, their strong binding also exerts a negative effect when the bound biotinylated glycopeptides are eluted from the streptavidin solid support. Harsh conditions are generally required for interrupting the strong interaction between biotin and streptavidin to release the bound glycopeptides from the solid support. However, the use of these harsh conditions can lead to decomposition of the glycopeptides resulting in low product recovery. This problem has recently been tackled by engineering azido-reactive reagents that have an easily cleavable linker between the biotin moiety and the azido-reactive moiety.

**Figure 5:**
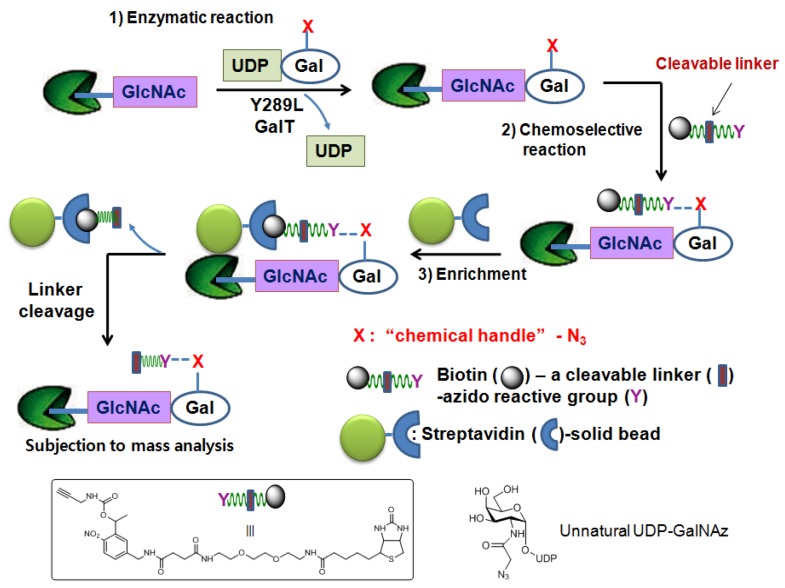
Chemoenzymatic tagging of *O*-GlcNAc-based enrichment strategieswith the photocleavable reagent.

This strategy is depicted in Figure 5. The same chemoenzymatic attachment approach is applied using UDP-GalNAz and mutant GalT. An engineered reagent consists of three important regions. The azido-reactive chemical region allows for its selective ligation to GalNAz- labeled proteins/peptides. The biotin region of the reagent facilitates enrichment of biotin-GalNAz-labeled proteins/peptides from other unmodified species by exploiting its affinity for streptavidin. Lastly, the readily cleavable linker region allows for the easy release of the isolated proteins/peptides from the solid support. Hart *et al.* have applied this strategy by using a biotin-photocleavable linker-alkyne reagent [85] which utilizes the ultraviolet light-catalyzed photochemical reaction for the release of the molecules of interest and identified O-GlcNAcylated sites on several neuronal proteins such as tau, synucleins, and methylCpG-binding protein 2 [85].

#### 2.1.4. β-Elimination followed by Michael Addition Chemistry-based enrichment approach

As the *O*-GlcNAc glycosidic linkage is susceptible to alkali-induced β-elimination, GlcNAc-eliminated peptides/proteins formed by alkali-induced β-elimination, followed by loss of water at Ser/Thr can provide modification site information [86]. Wells *et al.* have improved this method for easier mapping of the sites of O-GlcNAc modification. In this method, base-catalyzed β-elimination of *O*-GlcNAc results in α,β-unsaturated carbonyl followed by Michael Addition of a nucleophile such as dithiothreitol (DTT) (termed as “BEMAD”) to label the β-eliminated *O*-GlcNAc sites [51]. The DTT tag allows for enrichment via thiol-affinity chromatography. Unlike the *O*-GlcNAc moiety, as the DTT tag does not fall off during standard CID/CAD MS, it can provide the site information. Several *O*-GlcNAc sites of proteins such as synapsin I, nuclear pore complex proteins, actin, myosin and IRS-1 were mapped by this method [51]. However, the harsh conditions used in the BEMAD method can lead to degradation of peptides and side reactions with unmodified serine and threonine residues [87]. In addition, the inability of this method to differentiate *O*-GlcNAc from phosphate modification necessitates additional control experiments [88] and confirmation by independent methods.

In addition to the development of the enrichment strategies described above, recent advances in the MS field including the novel fragmentation methods make it possible to map *O*-GlcNAc with relatively small protein samples.

### 2.2. New MS Fragmentation Techniques

The *O*-GlcNAc glycosidic bond is much weaker than the peptide bond. Therefore, in standard CID/CAD MS, GlcNAc almost always falls off the peptide resulting in the loss of modification site information [56,57,89]. Furthermore, in CID/CAD MS, the *O*-GlcNAc peptide does not produce sufficient fragment ions, giving poor sequencing information. This arises from the loss of energy associated with the cleavage of the *O*-glycosidic bond. Recently, electron-capture dissociation (ECD) has been introduced as a new MS/MS fragmentation technique that uses low energy electrons to react with peptide captions in the magnetic field of a Fourier transform ion cyclotron resonance mass spectrometer [90]. Unlike CID/CAD MS, ECD does not necessarily break the weakest bond. In ECD, peptide fragments retain PTMs such as phosphorylation [91,92] and *O*-GlcNAc [52]. However, the high cost and experimental difficulties have prevented their widespread use. A similar MS/MS fragmentation technique called electron-transfer dissociation (ETD) has been developed which works well in cheaper ion trap mass spectrometers [93,94]. ETD uses radical anions rather than free electrons to transfer an electron to a protonated peptide and cleaves randomly along the peptide backbone while preserving the side chains, *O*-GlcNAc and other peptide modifications. This technique only works well for higher charge state ions (z > 2). However compared to CID, ETD renders more efficient fragmentation of longer peptides or even whole proteins, which makes this technique important for the proteomic analysis of *O*-GlcNAc modification. Recent applications of the ETD technique include the site mapping of *O*-GlcNAc on IRS-1 [95], Forkhead box O1 (FoxO1) [14], and peroxisome proliferator-activated receptor γ coactivator-1α (PGC-1α) [96]. By combining ETD MS/MS with enrichment methods, a number of *O*-GlcNAc sites on diverse proteins have been mapped [97].

### 2.3. Quantitative Proteomics of *O*-GlcNAc

As described above, the dynamic cycling of *O*-GlcNAc is sensitive to the cellular stimuli and the deregulated *O*-GlcNAc level is associated with several disease states. Therefore, quantitative analyses of *O*-GlcNAc levels on different proteins or at different states are necessary to understand the functional roles of *O*-GlcNAc in the specific cellular events at the molecular level.

#### 2.3.1. Identification and quantification of *O*-GlcNAc glycosylation *in vivo*

**S**table **i**sotope **l**abeling with **a**mino acids in **c**ell culture (SILAC) is an MS-based technique that detects differences in protein abundance among samples using stable (non-radioactive) heavy isotope labeling [97,98,99] and has become popular as a quantitative proteomic method. In this approach, proteins are metabolically labeled by culturing cells in media containing normal and heavy isotope amino acids. Metabolic incorporation of the amino acids into the proteins results in a mass shift that can be detected by a mass spectrometer. For example, two populations of cells are differentially incubated with light medium containing normal arginine (Arg-0) or heavy medium containing arginine labeled with six carbon-13 atoms (^13^C) (Arg-6) instead of normal carbon-12 (^12^C). All of the heavy arginine-containing peptides are 6 Da heavier than their normal counterparts. When both samples are mixed, pairs of chemically identical peptides of different stable-isotope composition are differentiated and the ratio of peak intensities in the mass spectrum reflects the relative protein abundance. The SILAC methodology has been used to test the effect of glycogen synthase kinase-3 inhibition on quantitative *O*-GlcNAc modification changes in COS7 cells [97]. Another cell culture labeling application of SILAC to study *O*-GlcNAc quantitatively was reported by Wells, *et al.* [98]. In this application, isotopically labeled glutamine (^15^N-Gln) was used to metabolically label cellular aminosugars such as GlcNAc, GalNAc, and sialic aicd with heavy nitrogen which enables differentiation of levels in different samples. The major disadvantages of SILAC include the high cost of the isotope-containing amino acids and its inability to study the *O*-GlcNAc modification of post-mitotic neurons or tissue samples because it requires multiple cell divisions for isotope incorporation.

#### 2.3.2. Identification and quantification of *O*-GlcNAc glycosylation *in vitro*

**Figure 6:**
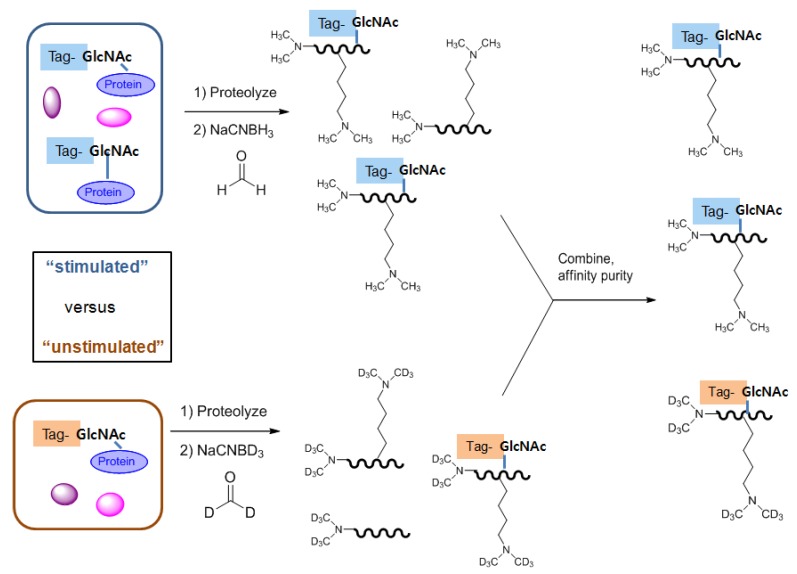
Quantitative isotopic and chemoenzymatic tagging (QUIC-tag) strategy.

Similar MS/MS spectrometry-based isobaric labeling strategies have been introduced for quantitative analysis of *O*-GlcNAc modification [102,103,104]. These strategies use isobaric labeling of peptides from different samples/treatments with different tags that consist of the mass reporter, mass balance, and peptide reactive regions. Lighter reporter regions are combined with heavier mass balance regions, such that the entire tag attached to the peptide adds the same mass shift. Therefore, after mixing, in MS^1^, (Ed- why the superscript when you have no notes?) the peptides appear as a single precursor. However, during MS^2^ the peptides are cleaved to produce sequence-specific fragment ions, which help to determine the peptide sequence as well as the different *m/z* reporter ions that provide quantitative information regarding the relative amount of the peptide in the samples. There are two types of isobaric tags: Tandem mass tags (TMT) [102,103] and isobaric tags for relative and absolute quantitation (iTRAQ) [104]. Recently, a similar peptide labeling method termed as quantitative isotopic and **c**hemoenzymatic tagging (QUIC-tag) has been introduced. This method combines chemoenzymatic tagging of O-GlcNAc proteins with a stable isotopic labeling strategy [105]. In this method, *O*-GlcNAc proteins from two different cell states (for example, stimulated *versus* unstimulated, diseased *versus* normal) are chemoenzymatically labeled, proteolyzed and differentially labeled with ‘light’ or ‘heavy’ isotopes via a modified dimethyl labeling method. The dimethyl labeling strategy uses either formaldehyde/NaCNBH_3_ or deuterated formaldehyde/NaCNBD_3_ to incorporate dimethyl or deuterated dimethyl into N-terminal amines and ε-amino groups of lysine residues by reductive amination. This creates mass differences of 6 × n between the peptides from the two cell populations, where n is the number of primary amine groups in the peptide. The resulting mixtures are combined, and *O*-GlcNAc peptides of interest are specifically enriched by affinity chromatography for selective quantification by LC-MS. The relative level of *O*-GlcNAc modification in the two cellular states is identified by calculation of the chromatographic peak area determined by the MS response to each eluting glycosylated pair of peptide ions (Figure 6). Using the QUIC-tag approach, several stimulation effects, including OGA inhibition on the dynamic change of *O*-GlcNAc in the brain, have been examined [106].

### 2.4. Significances of the Chemical Tools Designed for *O*-GlcNAc Proteomic Studies and Future Directions

Phosphorylation has long been considered a major regulatory mechanism in cellular processes. Aided by the various rapid and efficient strategies and the novel mass spectrometric techniques for *O*-GlcNAc proteome, nearly one thousand *O*-GlcNAc modified proteins have been identified and the number of identified *O*-GlcNAc sites has been greatly increased (for *O*-GlcNAc proteome information, see dbOGAP: Database of *O*-GlcNAcylated Proteins and Sites, http://cbsb.lombardi.georgetown.edu/hulab/OGAP.html). Many of these identified *O*-GlcNAcylated proteins are important regulatory proteins in cellular events, suggesting that *O*-GlcNAcylation is another major regulator of cellular signaling. Aided by recent advances in our ability to detect and quantitate the PTMs it is now known that the crosstalk between *O*-GlcNAcylation and phosphorylation exists at the modification site level and that the cycling dynamics of these two PTMs at the protein level are differentially regulated. For instance, phosphorylation of Sp1 transcriptional factor gradually rises to reach a peak around 4 h after insulin stimulation, whereas *O*-GlcNAcylation of Sp1 rapidly increases to reach a peak at 30 min, followed by a gradual decline, and returns low levels by 4 h after insulin treatment [107]. In order to better understand the modification effect on the protein level, continued efforts on *O*-GlcNAc proteomic studies, including the quantitative analysis of *O*-GlcNAc *in vivo*, should be invested. Once the modification sites of proteins of interest are determined, development of more direct research tools, such as site-specific *O*-GlcNAc antibodies, will be very helpful to progress our understanding of the interplay between *O*-GlcNAcylation and phosphorylation, and thereby significantly expand our view of signal transduction.

## 3. *O*-GlcNAc Regulatory Enzymes

The control of *O*-GlcNAc modifications of nuclear and cytoplasmic proteins provides a means of influencing numerous cellular events and the potential to manage various human diseases. An increasing awareness in the importance of the apparent functional interplay between *O*-GlcNAc and *O*-phosphate has prompted chemists/biochemists to develop useful tools to aid the *O*-GlcNAc modification. There are now several useful chemical and biochemical means available for the manipulation of the *O*-GlcNAc in culture cells or in living organism.

*O*-GlcNAc cycling involves only two enzymes: OGT [9,10,11] and OGA [12,13]. Therefore, selective and potent manipulation of *O*-GlcNAc in cells can be achieved by manipulation of these two enzymes’ activities. Small-molecule inhibitors of kinases have proven invaluable for studying the physiological roles of phosphorylation [108]. Cell permeable inhibitors provide several benefits over genetic methods in that there is no need for transfection reagents or viral infection, which renders this method especially useful in cells that are not efficiently transfected. Additionally, cells can be easily monitored according to the dose and time dependent manner and the reversibility of any effects on cells can be observed by removing inhibitors. The design and development of enzyme-specific inhibitors is based on a mechanistic understanding combined with the structural information of the enzyme catalytic sites. Structural and mechanistic studies of these enzymes can begin with the carbohydrate-active enzymes (CAZY) classification [109,110,111,112] because this classification is built on the basis of the amino-acid sequence similarities of the catalytic domains of enzymes and provides the structural and mechanistic insight on the enzymes of interest from the structurally or mechanistically known enzymes of other organisms in the same family. OGA and OGT are classified in CAZY families GH84 and GT41, respectively. As the first step toward the development of these small-molecule modulators, efficient screening methods for enzymes’ activity must be developed. This review discusses several assay methods that have been developed for the monitoring of enzymes’ activities and/or the screening of the compound libraries to discover potent inhibitors of the enzymes, and the significant contributions of these chemical tools to the evolution of our knowledge about the *O*-GlcNAc enzymes and their functional roles.

### 3.1. OGA

#### 3.1.1. Brief background

**Figure 7:**
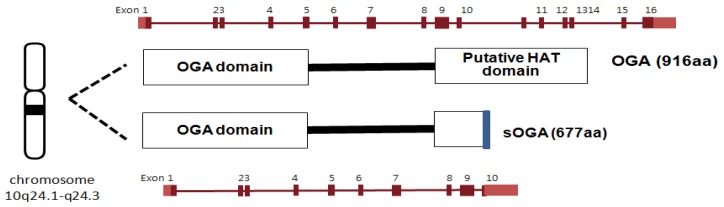
Human OGA isoforms.

OGA is a neutral hexosaminidase and takes the D-GlcNAc β-linked to Ser/Thr and liberates, through hydrolysis, β-D-GlcNAc giving net retention of anomeric configuration [12,13]. Neitherα-linked GlcNAc nor GalNAc is hydrolyzed by OGA. OGA and meninginoma expressed antigen 5 (*MGEA5*) were shown to be encoded by the same gene [113,114]. OGA has been mapped to chromosome 10q24.1-q24.3, which is a region associated with Alzheimer’s disease. The OGA gene is also implicated with the development of type II diabetes since single nucleotide polymorphism in the MGEA5 gene has been linked to the occurrence of type II diabetes in a Mexican population [115]. The OGA sequence is highly conserved in eukaryotic species, especially in mammals, but absent in prokaryotes and yeast [13]. The OGA gene encodes two alternatively spliced isoforms of OGA (Figure 7). The full length OGA is a 916 amino acid protein (103 kDa) which consists of the catalytic N-terminal domain and the putative acetyl transferase C-terminal domain linked through a highly disordered middle region of 150 amino acids [114,116,117]. Although the C-terminal domain of mouse OGA has been reported to have histone acetyltransferase (HAT) activity [118], more recent efforts have failed to support these findings [119]. The other isoform is a 677 amino acid protein that lacks the C-terminal third of the full-length OGA but contains an additional 15 amino acid residues [13,116,120]. According to cell fractionation studies, full length OGA predominantly localizes in the cytoplasm while the short OGA isoform resides in the nucleus [13,116,120].

#### 3.1.2. OGA activity assay methods and mechanistic studies of OGA

**Figure 8:**
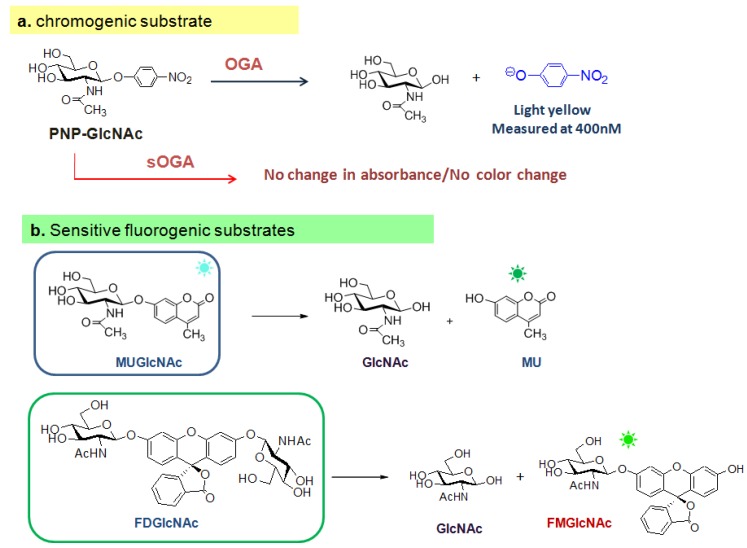
OGA activity assays using a chromogenic PNP-GlcNAc substrate (a) and two fluorogenic substrates (b).

The conventional *in vitro* assay for measuring OGA activity uses a chromogenic substrate, *p*-nitrophenyl 2-acetamido-2-deoxy-β-D-glucopyranoside (PNP-GlcNAc) [12], which is also used as a substrate for lysosomal hexosaminidases that are functionally related to OGA. Enzymatic activity is monitored spectrophotometrically by measuring the absorbance at 400 nm of a *p*-nitrophenolate ion that is enzymatically hydrolyzed from PNP-GlcNAc [12,121] (Figure 8a). However, this assay is relatively insensitive because the substrate undergoes only a modest change in absorbance upon cleavage and the enzymatic activity of short OGA is not detectable with PNP-GlcNAc. Recently, more sensitive assay methods involving fluorescence have been introduced to monitor OGA activity.

**Figure 9:**
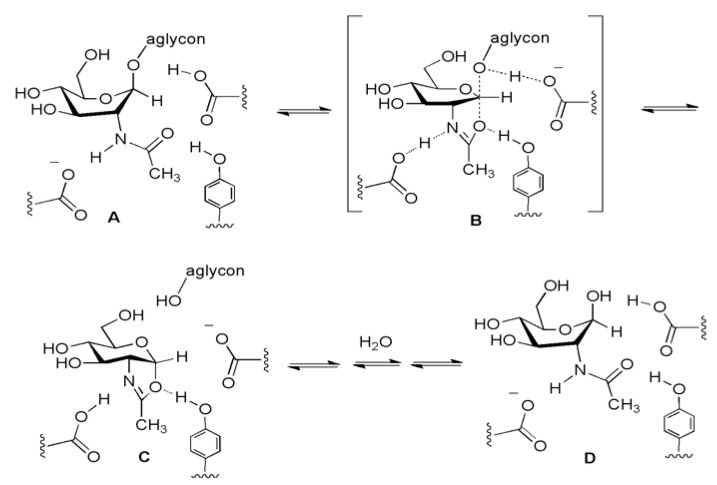
OGA uses the substrate-assisted catalytic hydrolysis mechanism (A→[B]‡→C→D).

The two fluorogenic substrates currently being used for *in vitro* OGA activity assay are4-methylumbellifery 2-deoxy-2-*N*-acetyl-β-D-glucopyranoside (MU-GlcNAc) [38] and fluorescein di(*N*-acetyl-β-D-glucosaminide) (FDGlcNAc) [41] (Figure 8b). MU-GlcNAc is hydrolyzed by the action of OGA to liberate 4-methylumbelliferon and the progress of enzyme reaction is determined by fluorescence measurements. Using this substrate and its derivatives, Macauley *et al.* have performed the first systematic study on the catalytic mechanism of OGA. In their mechanistic study, a series of substrates bearing different numbers of fluorine atoms substituted at the methyl group of the *N*-acetyl moiety was synthesized and examined to determine the effect of this substitution on the rate of catalysis [38]. Based on the decreased rate of OGA’s catalytic reaction with increasing number of fluorine substitutions decreased and the fact that substitution of electronegative fluorine atoms lowers the basicity of the carbonyl group and decreases the nucleophilicity of carbonyl oxygen, it has been proposed that OGA uses a substrate participation mechanism involving the acetamido group acting as a nucleophile. Such a trend was also observed for the human lysosomal hexosaminidases that have a catalytic mechanism involving substrate-assisted catalysis (Figure 9). However, the negative effect of the fluorine substitutions on the rate of enzymatic reaction is larger in lysosomal hexosaminidases than in OGA, which suggests that there is a steric factor difference in the catalytic center between them [38]. Thus, OGA is thought to have a relatively deep active site architecture that can tolerate some extension of the *N*-acetyl moiety of the substrate, whereas lysosomal enzymes have a shallow active pocket that does not allow more sterically hindered modification of *O*-GlcNAc. The key catalytic residues of human OGA have been suggested to be Asp174 and Asp175 [39]. Subsequent crystallographic studies of bacterial OGA homologues (*Clostridium perfringens* *Cp*GH84 [122,123] and *Bacteroides thetaiotaomicron* *Bt*GH84) [124] have provided further support for this “substrate-assisted” catalytic mechanism and for the structural difference of the active site architecture between OGA and lysosomal hexosaminidases. In addition, these kinetic and structural studies have also defined Asp174 and Asp175 as two general acid/base catalytic residues within the human OGA.

Although, the short and long OGA isoforms contain the identical N-terminal catalytic sites, no activity had been detected for several years in the short isoform *in vitro* with PUGNAc as an enzyme substrate. However, the use of a fluorogenic substrate, FDGlcNAc, revealed that both isoforms are active *in vitro* [41]. The FDGlcNAc substrate is non-fluorescent but becomes fluorescent when enzymatically hydrolyzed due to the generation of fluorescent, fluorescein-conjugated mono-GlcNAc (FMGlcNAc). Due to the greatly enhanced sensitivity of the assay, as well as the increased affinity of FDGlcNAc for the enzyme, the catalytic activity of short OGA isoform has been detected. Additionally, the short OGA has been shown to be active against cellular extracts, but at a much lower rate than the long isoform [41]. These *in vitro* data may suggest that the C-terminus of OGA is important for full enzymatic activity although the catalytic site of OGA lies in the N-terminus. Caspase-3, a member of the cysteine-aspartic acid protease (caspase) family that and serves as the “executioner protease” in apoptosis, cleaves the middle region of OGA *in vitro* and during apoptosis *in vivo* [119,120]. Cleavage of OGA by caspase-3 does not affect *O*-GlcNAc hydrolyzing catalytic activity. OGA activity is absent if the N-terminal region alone is overexpressed in cells, but fully present if this region is co-overexpressed with the C-terminal region. This observation further supports the proposal that an interaction between the N-terminal and C-terminal domains may be required for the optimal OGA activity. However, this does not exclude the possibility that the short OGA is as active as the long form *in vivo*, because there may be other factors, such as PTMs or binding adaptor proteins. The three key questions that remain unanswered are the nature of the functional role of the short OGA, the method by which *O*-GlcNAc cycling is regulated within the cell, and the function of the C-terminal domain.

**Figure 10:**
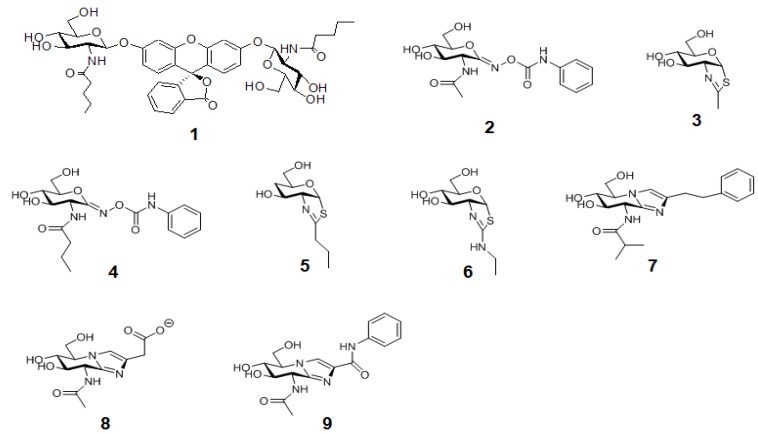
The structures of OGA-specific pentanamide FDGlcNAc substrate **1** analogueand OGAinhibitors discussed in the text.Compounds **2**,**3**,**8**,**9** are nonspecific OGA inhibitors, while **4–7** are OGA-specific inhibitors.

The second generation of an FDGlcNAc derivative (**1** in Figure 10) that is specific for OGA, but not for the functionally related lysosomal enzymes, has been developed [37] based on the structural insights on the catalytic center architectures of the enzymes. The main advantage of this OGA-specific fluorogenic substrate is its ability to enable the differential detection of OGA’s activity from that of other endogenous hexosaminidases, which facilitates the high-throughput analysis of OGA within cellular assays and serves as an imaging agent for the *in vivo* OGA analysis.

These aforementioned sensitive fluorogenic substrates have enabled the high-throughput analysis of enzyme activity, including inhibitor screening, using a multiwall plate format. The availability of rapid enzyme assays and insights on the mechanism and structure of OGA have facilitated the design of selective OGA inhibitors (more in reviews [43,44]). Briefly, previously known non-selective OGA inhibitors,*O*-(2-acetamido-2-deoxy-D-glycopyranosylidene)-amino-*N*-phenylcarbamate (PUGNAc, **2**) [13,125] and GlcNAc-thiazoline, **3** [38,126], have been modified to have the extended *N*-alkyl chains(Butyl-PUGNAc **4** and NButGT **5**), which can sit in the OGA’s active pocket but not in the more enclosed pocket of human HexA and B [37,38]. Another selective but still very potent inhibitor, “ThiametG” (**6**), has also been developed [127]. ThiametG is related to *N*-butyl thiazoline but contains an isothiourea moiety that renders a more favorable electrostatic interaction with the catalytic residue. In a similar vein, Dorfmueller and co-workers have developed a selective inhibitor, GlcNAcstatin (**7**) [40,128], which bears the identical tetrahydroimidazopyridine scaffold as the non-selective OGA inhibitor “Nagstatin”/sugar imidazole derivatives (**8**,**9**) [129,130,131], but contains a bulky isobutanamido moiety at C2 that offers selectivity for OGA.

### 3.2. OGT

#### 3.2.1. Brief background

**Figure 11:**
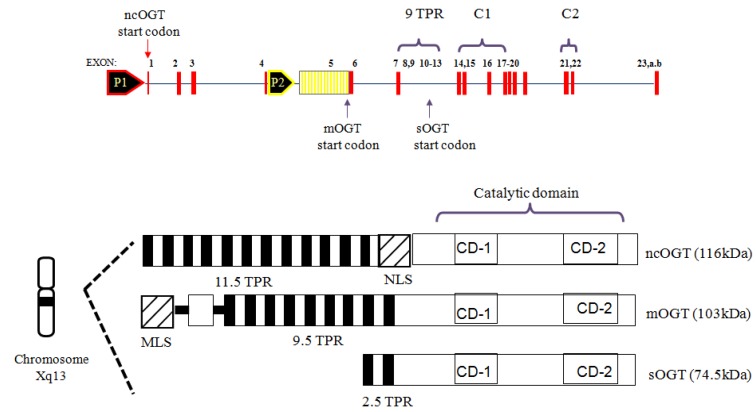
Human OGT isoforms.

As stated earlier, OGT transfers GlcNAc from α-linked UDP-GlcNAc to the hydroxyls of serine and threonine residues to form the β-linked *O*-glycosyl proteins [9,10,11]. OGT is classified as a family GT41 in the CAZY database [110]. OGT has numerous protein targets. To date, OGT has been found to glycosylate almost one thousand substrates and it is encoded by a single highly conserved gene in animals [10,13,132]. OGT is found in many organisms, from *Caenorhabditis elegans* to humans, and shares very high homology among species. The human OGT gene resides on the X chromosome (Xq13.1), which is a region associated with Parkinson’s disease [133,134]. Knockout studies have shown that OGT is required for the viability of embryonic stem cells and the loss of *O*-GlcNAc results in concomitant loss of cell function and eventually cell death [135]. However, OGT knockout in *C. elegans* does not lead to the death of the worm but produces profound metabolic changes, such as insulin resistance and carbohydrate storage [136]. For humans, there are three isoforms of OGT from alternative splicing, all of which consist of two distinct N-terminal and C-terminaldomains [10,134,137,138] (Figure 11). The N-terminal domain contains tetratricopeptide repeat (TPR) motifs, which are involved in protein-protein interaction. TPR motifs are highly conserved 34-amino acid repeats that are arranged as two α-helices that pack together to form a right handed super-helix with a large groove available for protein and ligand binding [139,140]. In contrast, the C-terminal domain is the catalytic domain that is identical in all three isoforms and has homology to glycogen phosphorylase [24,32,33,141]. Each OGT isoform has a unique N-terminus with different numbers of TPRs at the N-terminus. This unique N-terminus of each isoform is thought to contribute to their sub-cellular localization. Expression of each OGT isoform varies among different cell types and different tissues, suggesting that each isoform may have distinct functions and respond differently to cellular signaling depending on its tissue distribution [10,142]. The longest isoform has 11.5 TPR motifs, is found in the nucleus and cytoplasm (ncOGT; 116-kD), and is known to be associated with transcriptional repression [143], proteasomal inhibition [144,145], and stress tolerance [146]. The next longest isoform produces an N-terminal domain that contains a unique mitochondrial targeting sequence, followed by a membrane-spanning α-helix that directs the enzyme to the inner leaflet of the mitochondrial membrane [138]. This mitochondrial OGT (mOGT; 103-kDa) has 9.5 TPRs and may be associated with the activation of transcription factor Sp1 by mitochondrial superoxide overproduction [147]. The shortest form of OGT (sOGT; 70-kDa) contains 2.5 TPRs and similar to ncOGT is found in the nucleus and cytoplasm. sOGT is thought to have an anti-apoptotic function [148]. Each OGT isoform has been shown to have different peptide and protein preferences *in vitro* [142,149]. In addition, by the sequential removal of TPR segments to explore the roles of the TPR domain length in OGT substrate recognition, it has been suggested that OGT uses different TPR motifs to recognize specific protein substrates and that the substrate recognition is compromised by deletion; however, no catalytic activity is affected [132,150].

The first structural insights into the architecture of OGT were provided by the crystal structure of the TPR domain of human ncOGT by Jinek *et al.* in 2004 [151]. ncOGT exists as a homo-dimer with each monomer consisting of 23 anti-parallel α-helices that form a right-handed superhelix. This TPR structure shows an extended superhelical architecture in which the inner surface of the helix is lined with several conserved asparagine residues. This extended superhelical structure and the presence of the asparagine ladder resembles the armadillo repeats found in the structures of importin α [152] and β-catenin [153], which are involved in the recognition of nuclear localization signaling peptides. This structural similarity suggests that the considerable length of the TPR motifs provides an extensive scaffold that may accommodate many protein partners in many different orientations and binding modes, thereby explaining the absence of an explicit primary sequence motif for the *O*-GlcNAc modification.

#### 3.2.2. OGT activity assay methods and the structural insights on OGT

Aided by the high-throughput enzymatic assays available for OGA, the design of potent and selective inhibitors for OGA has flourished in recent years, as described above. However, the same cannot be said for OGT due to the complexity and inconvenience inherent in OGT assays. The lack of a rapid and simple method for continuously monitoring OGT activity has impeded the efficient discovery of potent OGT inhibitors. 

A conventional *in vitro* OGT activity assay [132] uses a radiolabeled sugar donor substrate such as UDP-[^14^C]-GlcNAc. In this method, the OGT activity is measured by quantitating radiolabeled GlcNAc incorporation into the protein substrate such as Nup62, a nuclear pore protein known to have multiple *O*-GlcNAc sites. However, this method involves a tedious procedure, high cost, and inevitable radiochemical waste, which render it unamenable for rapid analysis. To date, several non-radiometric assay strategies have been developed for monitoring OGT activity.

**Figure 12:**
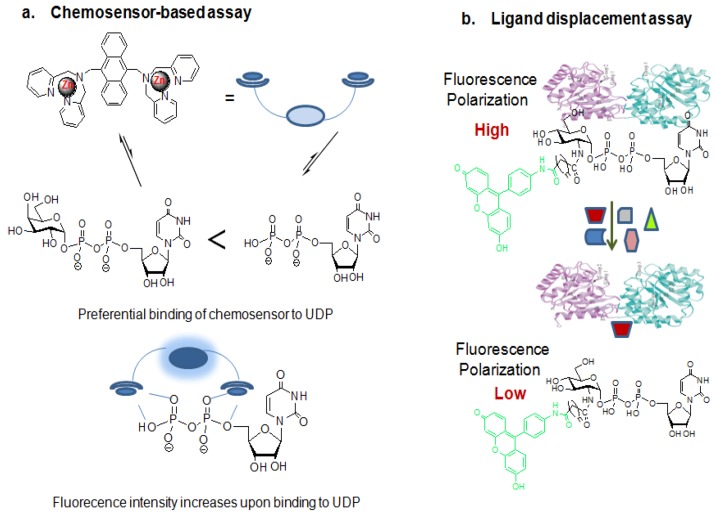
Strategies developed for OGT activity assay and high-throughput screening:(a)chemosensor-based glycosyltransferaseassayand (b) ligand displacement OGT assay.

The chemosensor-based glycosyltransferase assay (Figure 12a) has been introduced for high-throughput activity assay for a wide range of glycosyltransferases by the Hamachi group [154]. In this strategy, a glycosylated nucleotide such as UDP-glycoside is converted into the corresponding nucleotide by the action of a glycosyltransferase, and the amount of liberated nucleotide increases as the reaction proceeds. Since the nucleotide formation is equivalent to the progress of the glycosyl transfer reaction, sensing the generated nucleotide provides a means for identifying the progress of the enzyme reaction. This method utilizes an artificial fluorescent chemosensor that can allow for the distinction between a free nucleotide that is a product of the enzyme reaction and its corresponding glycosylated ones. The fluorescent chemosensor used in this strategy is a binuclear zinc complex-based, fluorescent probe (Figure 12a). This zinc-coordinated fluorescent probe binds to the free UDP more tightly than to the UDP-sugar. Therefore, the stronger binding multicomplex composed of the fluorescent probe and UDP produces a higher fluorescence intensity than the weaker binding multicomplex formed between the probe and the corresponding UDP-glycoside. The increase in fluorescence upon the coordination of the probe to UDP is considered the consequence of the suppression of photo-induced electron transfer quenching resulting from the phosphate-assisted coordination of the second Zn^2+^ atom. By measuring the difference in fluorescence intensity arising from the different binding affinity of this probe between the reactant (UDP-Gal) and the product (UDP), it is possible to calculate the enzyme activity. However, when this method is applied for the enzyme activity, special attention should be paid to the selection of the reaction system because the presence of any species in the reaction system that strongly binds to the chemosensor will significantly decrease the reaction sensitivity. For example, the phosphate or citrate buffers that are often used in enzymatic reaction buffers shouldn’t be used in this assay. Furthermore, the enzyme assayed should be purified, as otherwise some other substances in the cell lysates may quench or preferentially bind to the chemosensor, thereby harming the reaction sensitivity. In addition, the relative differences in the fluorescence intensity among the chemosensor probe, UDP and UDP-glycosides are relatively moderate.

A ligand displacement OGT assay (Figure 12b) [155,156] using fluorescent UDP-GlcNAc analogues and an active sOGT enzyme for the purpose of rapid screening of OGT inhibitors has been introduced by Walker *et al*. This strategy involves fluorescence anisotropy (fluorescence polarization), based on the idea that a fluorophore excited by polarized light will also emit polarized light. However, if a molecule is moving, it will emit the light at a different direction from the incident light and tend to “scramble” the polarization of the light. This “scrambling” effect is large when fluorophores are freely tumbling in solution but reduced when the tumbling rate of the fluorophores decreases. This method allows for the measurement of the protein interactions where one of the interacting partners is fused to a fluorophore. Binding of the fluorophore-containing molecule to an interacting protein leads to the formation of a larger and more stable complex that tumbles more slowly, in turn reducing the “scrambling” effect and increasing the polarization of the emitted light. In the fluorescent-based substrate displacement OGT assay, the binding of the fluorescent UDP-GlcNAc analogue to sOGT increases the anisotropy of this substrate analogue compared to its unbound state. Therefore, the amount of polarization observed is proportional to the amount of sOGT complex formed, which is itself proportional to the concentration of its binding to the sOGT partner. By measuring the difference in fluorescence polarization between the bound and unbound states of the substrate as a function of sOGT concentration, its dissociation constant or binding constant (~1.3 μM) was determined [156]. The displacement of this fluorescent analogue bound to sOGT by other non-fluorescent molecules decreases the fluorescence polarization. Therefore, addition of natural UDP-GlcNAc or UDP to apre-equilibrated mixture of sOGT and the fluorescent-labeled UDP-GlcNAc analogue replaces the fluorescent-labeled substrate from sOGT, thereby reducing the fluorescence polarization. The dissociation constants of the natural UDP-GlcNAc substance and the fluorescent-labeled UDP-GlcNAc analogue are similar (~1.5 μM for UDP-GlcNAc and ~1.3 μM for labeled UDP-GlcNAc analogue), implying that the fluorophore appendix on the substrate does not affect its binding onto the sOGT enzyme [156].

**Figure 13:**
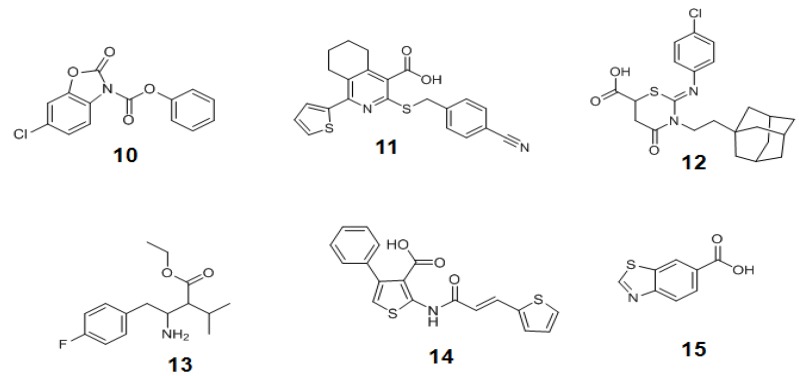
Structures of *in vitro* OGT inhibitors.

Using this strategy, a library of compounds has been screened and three structurally unrelated OGT inhibitors with the micromolar range inhibition (**10–12** in Figure 13) have been discovered. If these *in vitro* OGT inhibitors are able to reduce O-GlcNAc modification in cells, they can be useful tools to study the physiological role of *O*-GlcNAc. However, it has not been reported yet whether they are also active *in vivo* or not. Although this method allows for the rapid screening of the libraries of compounds, it does not provide the direct measurement of the glycosyl transferase activity of OGT, therefore necessitating other robust OGT activity assays to validate the positive hit compounds for OGT inhibition.

**Figure 14:**
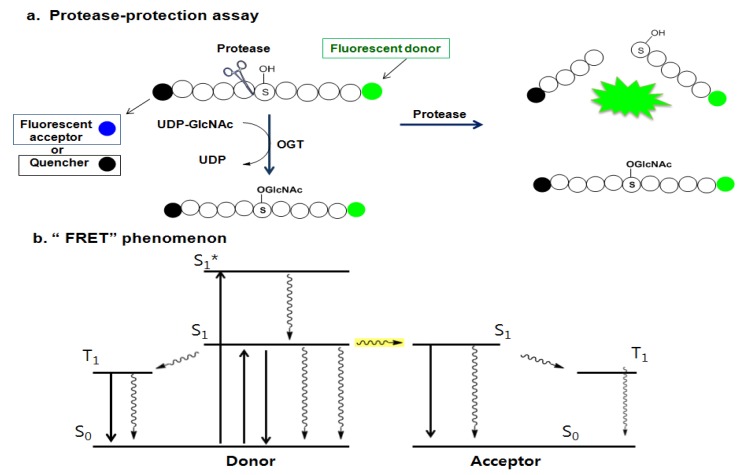
(a) Protease-protection assay: protease preferentially cleaves less sterically hindered unglycosylated peptide compared to the glycosylated peptide; and (b) the “FRET” phenomenon.

Later, Walker *et al*. presented another assay strategy called the protease-protection assay, which enables the monitoring of the glycosyl transferase activity of the enzyme by measuring the protease activity (Figure 14a) [157]. This assay exploits the “fluorescence resonance energy transfer (FRET)” phenomenon of a FRET pair tethered at each end of a peptide substrate. The FRET phenomenon begins when a fluorescent donor molecule absorbs the energy in a proper wavelength and is converted from its ground state S_o_ to an excited singlet state, S_1_*, and then to the relaxed singlet state, S_1_, by dissipating some of the absorbed energy. If there is no acceptor fluorophore near the donor, the relaxed singlet state (S_1_) of the donor returns to its ground energy state (S_o_) by emitting the remaining light energy at a longer wavelength in the form of specific fluorescence. However, if an acceptor fluorophore is present in the proximal distance to the donor, the energy emitted from the donor can be transferred into the acceptor fluorophore to activate the acceptor. Then the excited acceptor finally emits energy at a new wavelength through the same process described above. In order to accept the donor energy by a FRET acceptor, two criteria must simultaneously be satisfied: compatibility and proximity. In order to be a compatible acceptor its absorbance spectrum must overlap the emission spectrum of the donor molecule. If a compatible acceptor is close enough to the donor molecule for the energy to excite it, that molecule is suitable as a FRET acceptor (Figure 14b). In recent years, fluorescent acceptor molecules have been replaced with quencher molecules. Quenchers are chemically related to fluorophore acceptors but instead of emitting absorbed fluorescence resonance energy in the form of light, they transform the light energy to heat and hence remain dark. Dual-labeled constructs composed of fluorophore donor and quencher acceptor have no background fluorescence and thus greatly simplify many fluorescence-based assays. The substrate constructs Walker *et al*. employed for measuring OGT activity in their assay were specially designed peptide substrates with a fluorescein donor at one terminus and a quencher or fluorescent acceptor molecule at the other terminus. Their strategy is based on the observation that the cleavage of glycosylated proteins/peptides by protease is more difficult than that of non-glycosylated ones. The assay begins with O-GlcNAcylation of the specially designed peptide substrate labeled with a FRET pair by OGT. The O-GlcNAc-modified peptide is then treated with a protease that acts differentially on the glycosylated and the non-glycosylated peptides. The degree of peptide glycosylation, which is closely related to the amount of proteolysis, is determined by measuring the resulting FRET signal after proteolysis (Figure 14a). The OGT activity in this strategy is indirectly determined. If the presence of any factor affects the protease activity, the negative compounds can be judged as the positive hits. Therefore, other methods are required to validate this assay result. Using this method combined with the conventional radiolabeling OGT assay, three additional new OGT inhibitors (**13**–**15** in Figure 13) with a micromolar IC_50_ range (0.9 ~ 20 μM) have been discovered [157], but again, their use as OGT inhibitors *in vivo* has not yet been reported.

For a slightly different purpose, Bertozzi *et al*. has introduced a high-throughput method termed as the azido-enzyme-linked immunosorbent assay (azido-ELISA) [158]. This method has been developed for the interrogation of OGT’s substrate specificity and involves Staudinger ligation with azido-labeled substrate and a phosphine derivative. The assay uses an unnatural sugar donor, UDP-GlcNAz for OGT, to label the biotinylated peptide substrates that are captured onto avidin-coated 96-well plates, followed by chemoselective reaction with phosphine-FLAG. This FLAG-labeled epitope is then used for the colorimetric readout of horseradish peroxidase (HRP) activity by using the HRP-conjugated anti-FLAG antibody. This HRP activity readout data determines the relative OGT activity toward each peptide substrate. This approach facilitates the measurement of the OGT activity toward peptide substrates and the screening of various molecules for the OGT inhibitors. However, the whole process involves many reaction steps, is time consuming and increases the overall experimental errors. The results obtained from this method using the systematic variation of amino acids in the sequence of model peptide substrates suggest a modest preference of side-chains on both sides of the modification site, although OGT appears to have no evident primary sequence pattern of peptides to recognize. Therefore, there may be specific structural determinants underlying these substrate sequence preferences. Significant insights into the architecture of OGT have been provided by the X-ray structure of a bacterial OGT homolog, *Xanthomonas campestris* OGT, (*Xc*OGT) [159,160]. *Xc*OGT contains 5.5 TPRs followed by the GT domains that adopt the GT-B fold. The generic GT-B fold [112,161] consists of two β/α/β “Rossman” fold domains [catalytic domain I (CD-I) and catalytic domain II (CD-2)]. N-terminal CD-I binds the acceptor molecule while the C-terminal CD-II binds the nucleotide-sugar donor. Catalysis occurs at the interface of these two domains, carrying with the global domain conformational changes. The OGT enzyme is considered to operate via an inverting S_N_2-like reaction mechanism where the enzyme scaffold provides a general base that activates the incoming protein/peptide nucleophile (OH group of Ser/Thr) for the displacement of the leaving group (UDP portion of the donor) in a concerted manner.

### 3.3. Significances of the Chemical Tools Designed for Modulating the *O*-GlcNAc Enzymes and Future Directions

As aforementioned, aided by several useful, high-throughput, enzymatic assays and mechanistic and structural insights for OGA, several potent and selective OGA inhibitors have been developed and applied. The use of small-molecule OGA inhibitors has greatly assisted in the evolution of concepts about the roles of *O*-GlcNAc. In the vast majority of early studies of *O*-GlcNAc, *O*-GlcNAc was elevated by treatment of cells with inhibitors such as streptozotocin (STZ) [162] and PUGNAc [125]. These elevated *O*-GlcNAc levels have been linked to the development of insulin resistance (for examples with STZ see Ref. [163,164,165], and for PUGNAc see References [32,166]). Cell treatment with STZ causes apoptosis and pancreatic β-cell death [164,165,166,167,168]. A great body of data ascribes the apoptosis-inducing effect of STZ to the free radical NO production from the breakdown of the nitrosomoiety [169,170], but not to the selective inhibition of OGA. This reasoning is based on the fact that both PUGNAc and STZ cause increases in cellular *O*-GlcNAc levels, but only STZ causes celldeath [171,172]. PUGNAc is a very potent inhibitor of OGA, but it also strongly inhibits the human lysosomal enzymes, Hex A and B, which increases the ganglioside levels that could impact insulin resistance [173]. Due to the off-target effects, both STZ and PUGNAc may not be suitable chemical probes for the studies of OGA and *O*-GlcNAc. Treatment of 3T3-L1 adipocytes with a nonspecific inhibitor, PUGNAc, causes insulin resistance, whereas treatment of the cells with different OGA-specific inhibitors, show no effect on insulin desensitization [174,175]. In consistence with these results, recently, Vocadlo *et al*. have showed that treatment of rodents with a OGA-specific inhibitor does not cause insulin resistance although *O*-GlcNAc increases [176]. The link between the elevated *O*-GlcNAc level and insulin resistance in different cell lines and in different organisms should be thoroughly examined with these more specific chemical tools. Therefore, the application of these specific chemical tools promises exciting advances in this field.

In contrast to the availability of several useful specific OGA inhibitors, potent and selective OGT inhibitors are almost unknown. Clearly, intensive and continuous efforts focused on the development of simple and high-throughput OGT enzymatic assays and the design of the structure-inspired, OGT-specific inhibitors are essential to advance our understanding of the functional roles of OGT and *O*-GlcNAc cycling.

## 4. Conclusions

*O*-GlcNAcylation participates in many important cellular processes via its extensive and dynamic interplay with phosphorylation [29,30,35,36]. Consequently, dysregulation of the balance between these two modifications has been implicated in several diseases, such as cancer, diabetes, and neurodegenerative disease. Over the past several years, various chemical tools have been rapidly exploited to expand our understanding of the biological roles played by *O*-GlcNAc and have proven invaluable in elucidating our knowledge about cellular signaling. Nevertheless, important questions remain to be answered; one of the key questions is how OGT and OGA target so many substrates while retaining substrate specificity. Although it is now known that the substrate specificities of these enzymes can be determined, in part, by their transient interactions with many binding partners to form various substrate specific holoenzymes, an enhanced understanding of the molecular mechanisms by which they are regulated will remain a key focus for future research. Continuing efforts directed at enhancing the chemical arsenal with more direct and efficient chemical tools, such as the development of high-throughput yet robust OGT assays and isoform-specific OGA and OGT inhibitors, will lead to new discoveries about *O*-GlcNAcylation. In addition, the generation of site-specific *O*-GlcNAc antibodies and the elucidation of the enzymes’ crystal structures will be the subjects of future research to expand our understanding about the roles of *O*-GlcNAc. All of these tools will provide deeper insights into the cell signaling paradigm.
